# Epicardial Lipomatosis as a Cause of Constrictive Pericarditis

**DOI:** 10.1155/cric/1335322

**Published:** 2026-09-06

**Authors:** Punam Ajay Raval, John Otieno Odhiambo, Kevin Ombati, Barbara Karau, Leonard Mzee Ngunga

**Affiliations:** ^1^ Department of Internal Medicine, The Aga Khan University Hospital, Nairobi, Kenya, aku.edu; ^2^ Department of Radiology, The Aga Khan University Hospital, Nairobi, Kenya, aku.edu; ^3^ Department of Cardiology, The Aga Khan University Hospital, Nairobi, Kenya, aku.edu

## Abstract

Epicardial lipomatosis is a rare cause of constrictive pericarditis. There is a paucity of literature on the condition, and it is possible that it may be missed in contemporary clinical practice. Herein, we report two patients with epicardial lipomatosis who presented with traditional cardiovascular risk factors. After an initial suspicion of pericardial effusion, both patients underwent multimodality imaging with transthoracic echocardiography, computed tomography, and cardiac magnetic resonance imaging, which demonstrated diffuse epicardial fat. Conservative management was pursued in both cases with optimization of heart failure therapy and the dialysis strategy, resulting in clinical improvement.

## 1. Introduction

Constrictive pericarditis is an uncommon cause of heart failure characterized by impaired ventricular filling, resulting from a rigid, noncompliant pericardium. Although tuberculosis remains an important cause in low‐ and middle‐income countries, particularly in sub‐Saharan Africa, constrictive pericarditis may also develop secondary to previous cardiac surgery, mediastinal irradiation, connective tissue disorders, malignancy, viral pericarditis, or idiopathic chronic inflammation. Regardless of the underlying etiology, progressive restriction of diastolic filling results in elevated systemic venous pressures, impaired cardiac output, and symptoms of right‐sided heart failure.

Epicardial lipomatosis (EL) is a rare condition characterized by excessive accumulation of nonencapsulated adipose tissue surrounding the heart. In most patients, it is found as an incidental imaging finding without hemodynamic significance. However, exceptionally large epicardial fat deposits can mimic pericardial pathology or mechanically restrict cardiac filling to produce constrictive physiology. Because of its rarity, EL remains an underrecognized cause of constrictive pericarditis.

Multimodality imaging plays a central role in establishing the diagnosis. While transthoracic echocardiography (TTE) usually represents the first‐line investigation, computed tomography and cardiac magnetic resonance imaging provide superior tissue characterization, allowing accurate differentiation among fat, fluid, and soft tissue masses. We report two elderly female patients presenting with constrictive physiology secondary to diffuse EL, highlighting the diagnostic challenges, the role of multimodality imaging, and the individualized management strategies associated with this rare clinical entity.

## 2. Case 1

A female in her late 70s with a history of systemic arterial hypertension, asthma, and an ischemic stroke 8 years prior (baseline functional status—MRS 3) presented to a tertiary teaching hospital in Kenya in December 2023 with shortness of breath on exertion (NYHA II), bilateral lower limb swelling, facial swelling, and a persistent cough, progressive over an 8‐month period. There was, however, no orthopnea, paroxysmal nocturnal dyspnea (PND), palpitations, or chest pain. Her regular medications included aspirin 81 mg once a day, rosuvastatin 40 mg at night, amlodipine 5 mg twice a day, albuterol 90 mcg 1–2 puffs a day, vitamin D3 2500 IU once a day, escitalopram 10 mg once a day, metoprolol 50 mg twice a day, and omeprazole 20 mg once a day. A few months prior to her review, she was also taking melatonin 10 mg once a day, loratadine 10 mg once a day, and quetiapine 25 mg once a day, which had been held due to excessive drowsiness.

Her examination was positive for mild pedal pitting oedema, normal peripheral pulses, a normal jugular venous pulse, normal heart sounds, and reduced breath sounds at the lung bases with basal crackles. The abdominal examination did not show any organomegaly. A diagnosis of heart failure was made, and the patient was sent for appropriate testing.

On laboratory workup, she was found to have a hemoglobin of 12.5 g/dL, a platelet count of 428 × 10^9^/L, and a white cell count of 9 × 10^9^/L. Renal function tests showed a creatinine of 71 *μ*mol/L and blood urea nitrogen of 5 mmol/L. NT‐proBNP was normal at 169 pg/mL. An iron and lipid panel, extended electrolytes, vitamin D, uric acid, and urinalysis were normal. An electrocardiogram demonstrated normal sinus rhythm without significant conduction abnormalities.

**Figure 1:**
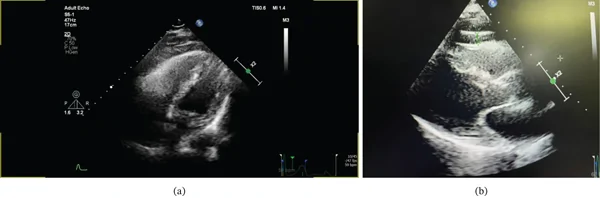
(a,b) 2D echocardiogram demonstrating a mild‐to‐moderate pericardial collection initially interpreted as pericardial effusion, mild concentric left ventricular hypertrophy, Grade I diastolic dysfunction, preserved left ventricular systolic function.

TTE demonstrated preserved left ventricular systolic function (LVEF 55%–60%), mild concentric left ventricular hypertrophy (interventricular septal thickness 13 mm, posterior wall thickness 11 mm), Grade I diastolic dysfunction, and normal biatrial size. A mild‐to‐moderate circumferential fibrinous‐appearing pericardial collection was identified and initially interpreted as a pericardial effusion. A septal bounce raised suspicion for constrictive physiology (Video S1). Tissue Doppler imaging demonstrated a medial e ^′^ velocity of 4.45 cm/s and a lateral e ^′^ velocity of 6.48 cm/s, whereas the inferior vena cava measured 16 mm with inspiratory collapse to 8 mm. Global longitudinal strain analysis was not available as part of the original echocardiographic study (Figure 1a,b).

Given the apparent fibrinous pericardial effusion and the high regional prevalence of tuberculous pericarditis, tuberculous constrictive pericarditis was initially suspected; however, sputum GeneXpert testing was negative.

Given the limited tissue characterization of TTE, diffuse epicardial fat could not be confidently distinguished from fibrinous pericardial fluid, prompting further evaluation with cross‐sectional imaging.

**Figure 2:**
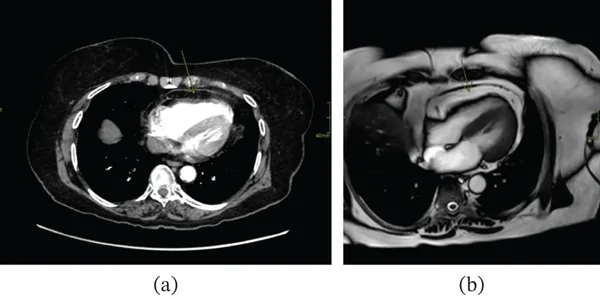
(a) Computed tomography (CT) of the chest showing findings suggestive of epicardial and mediastinal lipomatosis. (b) Cardiac MRI showing diffuse epicardial lipomatosis.

Subsequent CT scan of the pericardium showed fat stranding within the pericardium, also seen within the mediastinum (Figure 2a). Cardiac MRI was done. It demonstrated normal indexed biventricular volumes and function (LVEF 66%, RVEF 62%) and diffuse EL with mild‐to‐moderate mitral regurgitation and normal native T1 values (Figure 2b).

A final diagnosis of EL was made and she was commenced on medical therapy.

She was commenced on telmisartan 80 mg daily, eplerenone 25 mg daily, and torsemide 10 mg daily and was continued on metoprolol 50 mg daily with clinical improvement in symptomatology.

An option of surgical intervention was given if the patient′s symptoms did not improve. Long‐term imaging follow‐up was not available because the patient continued routine outpatient follow‐up locally, with emphasis on clinical symptom monitoring. Invasive hemodynamic assessment by cardiac catheterization was discussed but declined by the patient. Given the concordant clinical presentation and multimodality imaging findings demonstrating diffuse EL with constrictive physiology, further invasive evaluation was not pursued.

## 3. Case 2

The second case is of a female in her 80s on follow‐up for end‐stage renal disease on thrice‐weekly dialysis, hypertension, Type II diabetes, hypothyroidism, and heart failure. She also had a history of coronary artery disease, with a diagnostic angiogram revealing a 50% stenotic lesion in the left anterior descending artery. Her regular medications included amlodipine 5 mg once a day, atorvastatin 40 mg at night, sevelamer 800 mg once a day, levothyroxine 125 mcg once a day, trimetazidine 35 mg once a day, carvedilol 12.5 mg twice a day, gabapentin 25 mg once a day, isosorbide mononitrate 30 mg once a day, furosemide 40 mg once a day, and linagliptin 5 mg once a day.

She presented as a referral to the cardiology clinic due to episodes of intradialytic hypotension, necessitating stopping of dialysis or reduction in ultrafiltration. She reported occasional headache, dizziness, and fatigue. Her examination was positive for mild pedal pitting oedema, normal peripheral pulses, a normal jugular venous pulse, normal heart sounds, and reduced breath sounds at the lung bases with basal crackles. The abdominal examination did not show any organomegaly. An electrocardiogram done showed sinus rhythm, with no Q waves but poor R wave progression.

**Figure 3:**
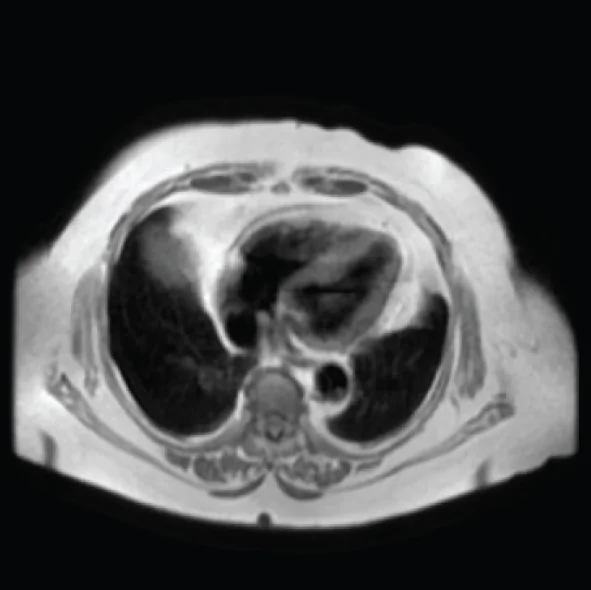
Cardiac MRI demonstrating prominent epicardial fat consistent with epicardial lipomatosis.

A 2D echocardiogram showed normal left ventricular systolic function. There was, however, a septal bounce to suggest constrictive physiology. On further assessment, there appeared to be epicardial fluid, with a differential of fat (lipomatosis). The right ventricle also appeared to collapse during the early diastolic phase. A differential of EL was made with constrictive physiology and collapse of the heart right ventricle during dialysis and subsequent hypotension. A cardiac MRI was done, confirming prominence of epicardial fat in keeping with EL (Figure 3). A plan was made for dialysis sessions to be conducted with the administration of norepinephrine at 0.1 mcg/kg/min to maintain normal intradialytic blood pressures. The patient declined any invasive interventions thereafter and continued to be managed conservatively.

## 4. Discussion

EL is a type of cardiac lipomatosis defined by the accumulation of mature, nonencapsulated fat tissue in the epicardial region, caused by hyperplasia of lipocytes. Although its etiology is unclear, it seems to be more common in older, obese people, with a female preponderance [1]. Its appearance on echocardiography can lead to misdiagnosis due to its similarity to other pathologies, and cardiac MRI is a crucial tool to accurately identify this condition [2]. It is usually benign but can rarely cause complications such as cardiac tamponade. Usually, epicardial fat is found in the atrioventricular and interventricular grooves, and some clues can be useful to delineate epicardial fat from pathology on TTE [1]. However, it can be challenging to rule out pathology in certain cases with TTE appearance of lipomatosis. Chest CT without contrast is a useful supplementary tool due to its excellent spatial resolution and ability to define fat. Cardiac magnetic resonance imaging, with its distinct tissue characterization ability, can distinguish between tissue types and verify that the epicardial mass is fat, as seen in the cases above [3].

Differentiating epicardial fat from pericardial effusion using TTE may be challenging. Epicardial fat typically appears as a relatively echogenic or speckled layer located predominantly anterior to the right ventricle, whereas pericardial effusion is usually echo‐free and circumferential [4]. However, these distinguishing characteristics may be obscured in patients with diffuse EL, fibrinous collections, or suboptimal acoustic windows, leading to diagnostic uncertainty. In our first patient, the circumferential fibrinous‐appearing collection was initially interpreted as pericardial effusion because echocardiography could not reliably differentiate diffuse adipose tissue from fluid, prompting further imaging.

Computed tomography provides excellent tissue characterization through attenuation measurements, with adipose tissue demonstrating characteristic low attenuation values (approximately −80 to −120 HU). Cardiac magnetic resonance imaging further improves diagnostic confidence through superior tissue characterization and differentiation of adipose tissue from pericardial fluid and soft tissue masses.

Although tissue Doppler imaging in our first patient did not demonstrate all of the classical Doppler features of constrictive pericarditis, including preserved or increased medial e ^′^ velocity and annulus reversus, constrictive physiology should not be diagnosed using a single echocardiographic parameter in isolation. Rather, diagnosis relies on integration of clinical presentation, ventricular interdependence, septal bounce, and complementary multimodality imaging [5]. In our patient, diffuse EL identified on CT and cardiac MRI provided the anatomical explanation for the observed constrictive physiology.

Restrictive cardiomyopathy was also considered in the differential diagnosis because of the patient′s age and evidence of diastolic dysfunction. Despite mild concentric left ventricular hypertrophy, preserved biventricular systolic function, normal biatrial dimensions, and demonstration of diffuse epicardial adipose tissue causing external cardiac constraint favored constrictive physiology rather than intrinsic myocardial disease [3].

In 1969, Kluge noted that EL could result in arrhythmia. Since then, several case reports have suggested that it may lead to various rhythm disturbances, including P‐wave abnormalities, atrial fibrillation, and even sudden death [6]. Malignant cardiac arrhythmias can arise from significant bleeding into the lesion. Arrhythmias may also result from atherosclerotic coronary artery disease, which is commonly found in affected individuals. Furthermore, it is believed that when the interatrial septum and right atrial wall are involved, particularly in patients with large lesions, the structure of atrial myocytes may be disrupted, potentially affecting the conduction pathways [7].

Although uncommon, constrictive physiology secondary to excessive epicardial fat has previously been described. Do et al. [8] reported a patient with massive epicardial fat volume producing hemodynamically significant constrictive pericarditis requiring surgical intervention, whereas Noly et al. [9] described giant pericardial lipoma causing severe cardiac constriction successfully treated with surgical resection. These reports, together with our cases, suggest that epicardial adipose tissue should be considered in the differential diagnosis of unexplained constrictive physiology, particularly when conventional causes are absent.

Owing to the rarity of EL, there are no evidence‐based guideline recommendations regarding optimal management. Treatment should therefore be individualized according to symptom burden, hemodynamic compromise, and surgical risk. Conservative treatment consisting of optimization of heart failure therapy, management of cardiovascular risk factors, and close clinical surveillance may be appropriate for patients with mild symptoms or prohibitive operative risk. Surgical debulking or pericardiectomy has been reported in isolated cases with severe compressive symptoms; however, these procedures are technically challenging because of the intimate relationship between epicardial fat and the coronary arteries, with high risk of injury [10]. A multidisciplinary strategy should be deployed in each case with long‐term follow‐up.

## 5. Conclusion

EL is an uncommon but important cause of constrictive physiology and may closely mimic fibrinous pericardial effusion on TTE. These cases highlight the importance of maintaining a broad differential diagnosis when evaluating patients with suspected constrictive pericarditis and demonstrate the complementary role of multimodality imaging, particularly CT and cardiac MRI, in establishing the correct diagnosis. Increased recognition of this rare entity may prevent misdiagnosis, avoid unnecessary investigations, and facilitate appropriate individualized management. Our report adds to the limited published literature by describing two elderly patients managed conservatively using multimodality imaging rather than surgical intervention.

## Funding

No funding was received for this manuscript.

## Consent

All the patients allowed personal data processing, and informed consent was obtained from all individual participants included in the study.

## Conflicts of Interest

The authors declare no conflicts of interest.

## Supporting information


**Supporting Information** Additional supporting information can be found online in the Supporting Information section. Video S1: 2D echocardiogram showing septal bounce.

## Data Availability

Data sharing is not applicable to this article as no datasets were generated or analyzed during the current study.
